# Carry-over effects of dry period heat stress on the mammary gland proteome and phosphoproteome in the subsequent lactation of dairy cows

**DOI:** 10.1038/s41598-022-10461-z

**Published:** 2022-04-22

**Authors:** Amy L. Skibiel, Jin Koh, Ning Zhu, Fanchao Zhu, Mi-Jeong Yoo, Jimena Laporta

**Affiliations:** 1 Department of Animal, Veterinary and Food Sciences, University of Idaho, Moscow, ID 83844 USA; 2 Interdisciplinary Center for Biotechnology Research, Proteomics and Mass Spectrometry Core, University of Florida, Gainesville, FL 32611 USA; 3 Department of Biology, Clarkson University, Potsdam, NY 13699 USA; 4 Department of Animal and Dairy Sciences, University of Wisconsin-Madison, Madison, WI 53715 USA

**Keywords:** Molecular biology, Physiology

## Abstract

Exposure to heat stress during a cow’s dry period disrupts mammary gland remodeling, impairing mammary function and milk production during the subsequent lactation. Yet, proteomic changes in the mammary gland underlying these effects are not yet known. We investigated alterations in the mammary proteome and phosphoproteome during lactation as a result of dry period heat stress using an isobaric tag for relative and absolute quantitation (iTRAQ)-based approach. Cows were cooled (CL; *n* = 12) with fans and water soakers in a free stall setting or were heat stressed through lack of access to cooling devices (HT; *n* = 12) during the entire dry period (approximately 46 days). All cows were cooled postpartum. Mammary biopsies were harvested from a subset of cows (*n* = 4 per treatment) at 14, 42, and 84 days in milk. Overall, 251 proteins and 224 phosphorylated proteins were differentially abundant in the lactating mammary gland of HT compared to CL cows. Top functions of differentially abundant proteins and phosphoproteins affected were related to immune function and inflammation, amino acid metabolism, reactive oxygen species production and metabolism, tissue remodeling, and cell stress response. Patterns of protein expression and phosphorylation are indicative of increased oxidative stress, mammary gland restructuring, and immune dysregulation due to prior exposure to dry period heat stress. This study provides insights into the molecular underpinnings of disrupted mammary function and health during lactation arising from prior exposure to dry period heat stress, which might have led to lower milk yields.

## Introduction

Global climate change is substantially reducing the profitability of dairy operations. Heat stress in lactating cows is estimated to cost the United States dairy industry more than $1 billion dollars annually due to cow morbidity, mortality, and milk loss^1^. High ambient temperature and humidity push cows beyond the upper limit of their thermoneutral zone, inducing behavioral and physiological adaptations that prioritize thermoregulation and survival while depressing heat-generating processes, such as food consumption, rumination, and milk synthesis^2–4^. As a result, heat stressed dairy cattle have a higher incidence of disease, lower fertility, and significantly lower milk yield relative to thermoneutral herd-mates^5,6^. Dairy cows are not only vulnerable to thermal stress while lactating, but also in the dry period preceding lactation. Cows heat stressed when dry have altered postabsorptive metabolism and produce 5 to 7.5 kg/day less milk during the subsequent lactation, even if they are actively cooled postpartum^7–11^. Thus, environmental heat stress has carry-over effects on the physiology and performance of dairy cows, extending beyond the period of exposure. Heat stress during the dry period may translate into an additional $810 million in annual milk losses in the U.S.^12^.

The dry period is a non-lactating period between successive lactations and is initiated in the last 6–8 weeks before the cow’s expected parturition. The dry period consists of an initial involution phase characterized by greater rates of mammary epithelial cell (MEC) apoptosis and a redevelopment phase where cell proliferation predominates^13^. The existence of a dry period allows for greater milk production in the subsequent lactation due to the replacement of non-functional, senescent MEC with a robust population of new cells^14^. During lactation, the capacity of the mammary gland to synthesize milk is dependent on MEC activity and number, the latter of which is driven by dynamic changes in the relative rates of apoptosis and cell proliferation across the lactation period^15–17^.

Heat stress has adverse impacts on cellular processes, such as apoptosis and cell proliferation, in the mammary gland and on mammary tissue microstructure. Heat stressed dry cows have lower mammary cell proliferation during the redevelopment phase of the late dry period^10^, and during the subsequent lactation, the mammary glands are comprised of fewer alveoli surrounded by larger areas of stromal connective tissue relative to cows that are actively cooled when dry^18^. In vitro studies have reported impairments in mammary development and function associated with hyperthermic culture conditions. After 8 h of incubation at high temperature, bovine MEC growth was stunted and branching morphogenesis and ductal extension halted^19^. Subjecting bovine MEC to high temperatures for as little as 30 min to 1 h decreased cell viability and cell cycle progression and induced cell apoptosis^20,21^.

Changes in molecular pathways in response to heat exposure have also been explored. Shortly after heat stress initiation, bovine MEC show an elevated expression of genes and proteins involved in the stress response and cell repair and downregulation of genes and proteins associated with the cell cycle, cell differentiation, cell structure, and macronutrient synthesis^19,21,22^. These results were not replicated in an in vivo study of heat stressed dry cows, which did not detect changes in expression of several genes involved in milk synthesis^23^. However, through RNA sequencing of mammary tissue, Dado-Senn et al.^24^, found that heat stressed dry cows downregulate genes involved in mammary parenchymal development and upregulate genes involved in the cell stress response, similar to results of in vitro experiments. Lastly, an in vivo study of acute heat stress in dairy cows found a higher abundance of proteins involved in mammary remodeling and a lower abundance of proteins involved in the TCA cycle and aerobic metabolism relative to pair-fed thermoneutral cows^25^. These studies have contributed to our knowledge of the cellular and molecular events occurring in mammary tissue after heat stress exposure, yet the impact of heat stress during the dry period on mammary protein expression in the subsequent lactation is currently unknown.

The objectives of this study were to investigate (1) the effects of prior heat stress exposure (i.e., during the dry, non-lactating phase of the production cycle) on the mammary proteome and phosphoproteome at key stages of the subsequent lactation and (2) the associated functional pathways and protein and phosphoprotein networks affected by dry period heat stress. We hypothesized that proteins involved in milk biosynthesis, cell differentiation and proliferation, and protein metabolism would be less abundant whereas proteins involved in cell death and cell stress responses would be more abundant in the lactating mammary gland as a consequence of exposure to dry period heat stress. Further, we expected that the phosphorylation status of proteins involved in these pathways will be altered by previous exposure to heat stress.

## Results

### Milk yield and components

Daily milk yield was higher in the CL cows relative to the HT cows (Table 1). Fat percentage was higher in HT compared to CL cows (Table 1). However, due to the greater daily milk yield of CL cows, both protein and fat yield were higher in CL cows compared to HT (Table 1).

**Table 1:** Milk yield and composition.

Variable	HT	CL	*P*-value
Milk yield (kg/day)	34.27 ± 0.73	39.93 ± 0.87	< 0.0001
Fat (%)	3.77 ± 0.03	3.52 ± 0.03	< 0.0001
Protein (%)	2.79 ± 0.02	2.90 ± 0.02	< 0.001
Fat yield (kg/day)	1.28 ± 0.02	1.38 ± 0.03	< 0.01
Protein yield (kg/day)	0.95 ± 0.02	1.15 ± 0.02	< 0.0001

Cows were either cooled (CL, access to fans and water soakers) or heat stressed (HT, no access to cooling devices) during the dry period (approximately 46 days pre-calving) and mammary tissue collected in their subsequent lactation, when all cows were managed as a single group and cooled. Data are presented as LSM ± SEM.

### Mammary gland proteomics

**Figure 1:**
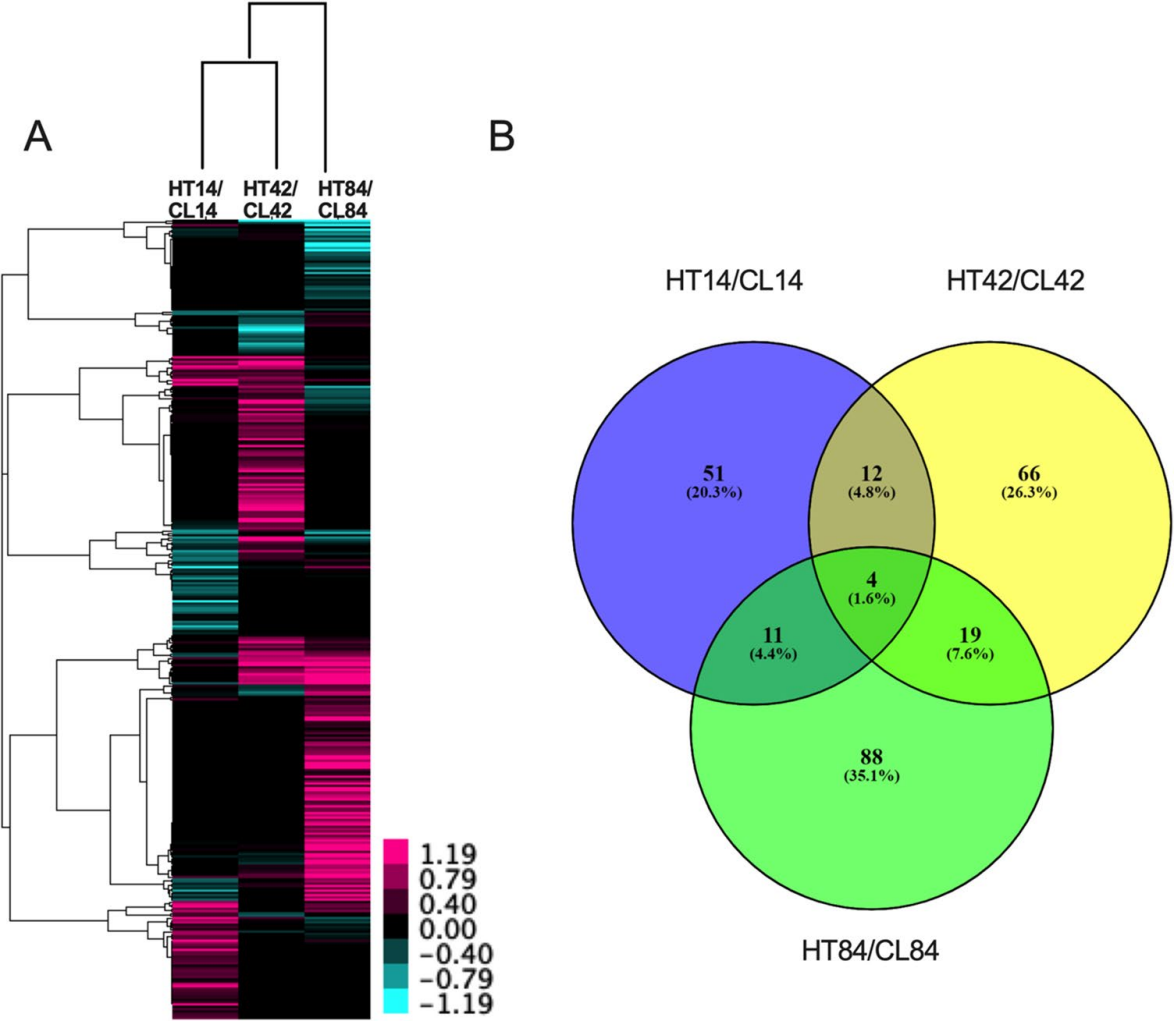
iTRAQ proteomics analysis of the mammary gland of dairy cows at three time points during lactation (14, 42, and 84 DIM). Cows were either cooled (CL, *n* = 4, access to fans and water soakers) or heat stressed (HT, *n* = 4, no access to cooling devices) during the dry period (approximately 46 days pre-calving) and mammary tissue collected in their subsequent lactation, when all cows were managed as a single group and cooled. (**A**) Heat map displaying unsupervised hierarchical clustering of proteins. Bars represent log_2_-fold changes in protein abundance between CL and HT at each time point. Magenta and cyan colors represent increased or decreased proteins in HT relative to CL, respectively (**B**) Venn diagram showing overlap of the number of differentially abundant proteins identified by iTRAQ proteomics. Proteins were considered differentially abundant between treatments at *P*-value < 0.05 and fold change > 1.3 or < 0.7.

Across all three time points and treatments, a total of 4694 proteins were identified (Supplementary Table S1). Of these, 4259 proteins were annotated, and 3673 proteins could be categorized based on cellular location and function using IPA. Most proteins were located in the cytoplasm (*n* = 1923) and function as enzymes (*n* = 882) (Supplementary Fig. S2). Based on our cutoff criteria (*P* < 0.05, fold change < 0.7 or > 1.3), 251 proteins were differentially abundant in the mammary glands of HT versus CL cows. Across all time points, more differentially abundant proteins were increased than decreased in HT relative to CL (Fig. 1A). The largest number of differentially abundant proteins occurred at 84 days in milk (DIM). Only the following four proteins were differentially abundant at all three time points (Fig. 1B): tubulin beta-5 chain (TBBM), cationic trypsin (TRY1), globin A1 (HBB), and prothymosin alpha (PTMA). Notably, TBBM, TTRY1, and HBB were increased at all time points, while PTMA was increased at 14 and 84 DIM and decreased at 42 DIM in HT relative to CL.

**Figure 2:**
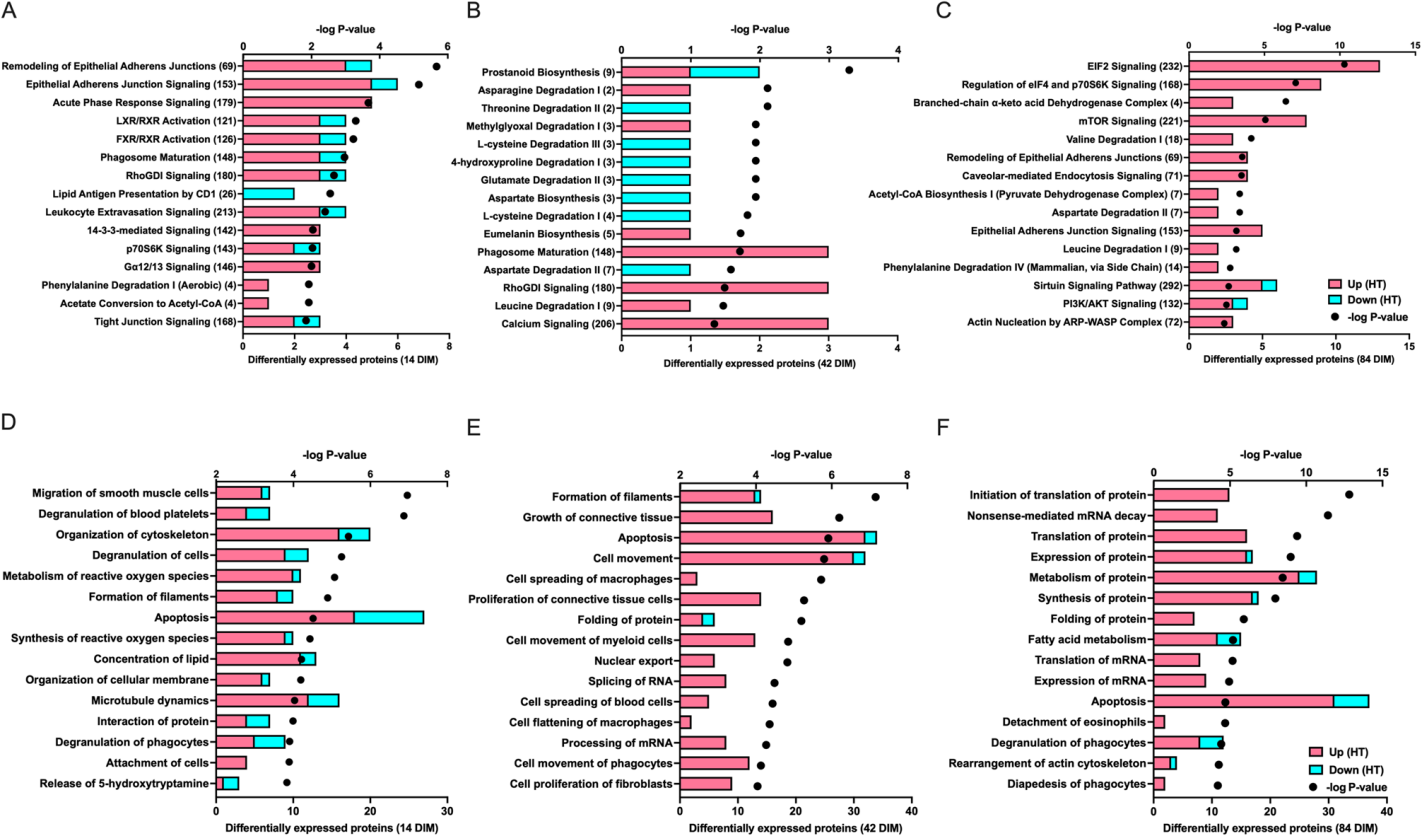
Top 15 pathways (**A**–**C**) and biological functions (**D**–**F**) of differentially expressed proteins in the lactating mammary gland of cooled and heat stressed cows. Cooled cows (CL, *n* = 4) had access to fans and water soakers whereas heat stressed cows (HT, *n* = 4) did not have access to cooling devices during the dry period (approximately 46 days pre-calving). Mammary biopsies were collected in the subsequent lactation at 14, 42, and 84 DIM, when all cows were managed as a single group and cooled. Enrichment analysis was performed by Qiagen Ingenuity Pathway Analysis (IPA, QIAGEN Inc., https://www.qiagenbioinformatics.com). Numbers in parentheses indicate the total number of molecules in the pathway in the IPA database. Upregulation or downregulation refers to protein expression in HT cows relative to CL cows.

Top pathways and functions affected by dry period heat stress at 14 DIM were related to immune function and the inflammatory response (e.g. *degranulation of platelets and phagocytes, acute phase response, leukocyte extravasation*), cell organization and structure (e.g. *cytoskeleton, microtubule dynamics, organization of cell membrane, formation of filaments, adherens junctions, RhoGDI signaling*), and synthesis and metabolism of reactive oxygen species (ROS) (Fig. 2A,D, Supplementary Table S2). Differentially abundant proteins at 14 DIM involved in immune function included proteins such as, C-reactive protein (CRP), prothrombin (F2), and serotransferrin (TF), which were more abundant in HT than CL, and prosaposin (PSAP), alpha-1-B glycoprotein (A1BG), and CD9 antigen (CD9), which were less abundant in HT than CL. Proteins in cell organization pathways were largely more abundant in HT relative to CL at 14 DIM (i.e. α-actinin (ACTN1) and canx protein (CANX)). Approximately 10 proteins enriched for ROS synthesis and metabolism functions were differentially expressed at 14 DIM and the majority of these proteins, such as glutamyltransferase 2 (TGM2) and Rho GDP-dissociation inhibitor 1 (ARHGDIA), were more abundant in HT compared to CL.

At 42 DIM, proteins involved in immune function (e.g. *spreading of macrophages, flattening of macrophages, phagosome maturation*), amino acid metabolism (e.g. *asparagine degradation, threonine degradation, L-cysteine degradation*), and cell structure/proliferation (e.g. *formation of filaments, growth of connective tissue*) were differentially abundant between HT and CL (Fig. 2B,E, Supplementary Table S2). Approximately 20 proteins enriched for immune pathways and functions were more abundant in HT than CL at 42 DIM. Some of these proteins included, dynactin subunit 2 (DCTN2), β-2 microglobulin (B2M), and vitronectin (VTN). Differentially abundant proteins at 42 DIM in the amino acid metabolism pathways included, aspartate aminotransferase (GOT2) and 2-amino-3 ketobutyrate coenzyme A ligase (GCAT), which were both less abundant in HT relative to CL, and l-asparaginase (ASRGL1) and isovaleryl-CoA dehydrogenase (IVD), which were more abundant in HT compared to CL. With the exception of prostaglandin D2 synthase (PTGDS), all differentially expressed proteins at 42 DIM with cell structure and proliferation functions were more abundant in HT relative to CL (e.g. tropomyosin β chain (TPM2), kininogen (KNG1)).

Top pathways and functions affected by differentially abundant proteins at 84 DIM were associated with protein metabolism (e.g. *translation, expression, metabolism, and synthesis*) and cell organization and structure (e.g. *actin cytoskeleton, adherens junctions*) (Fig. 2C,F, Supplementary Table S2). Several differentially abundant proteins in the protein metabolism pathways at 84 DIM were ribosomal proteins, such as 40 s ribosomal protein S5 (RPS5) and 40 s ribosomal protein S9 (RPS9) and were more abundant in HT relative to CL cows. Proteins involved in cell organization and structure included, transforming protein RhoA (RHOA), tubulin β chain (TUBB2A), and integrin β-1 (ITGB1, which were more abundant in HT than CL. Common affected pathways and functions across all three time points included immune function, cell structure and organization, and apoptosis.

### Mammary gland phosphoproteomics

**Figure 3:**
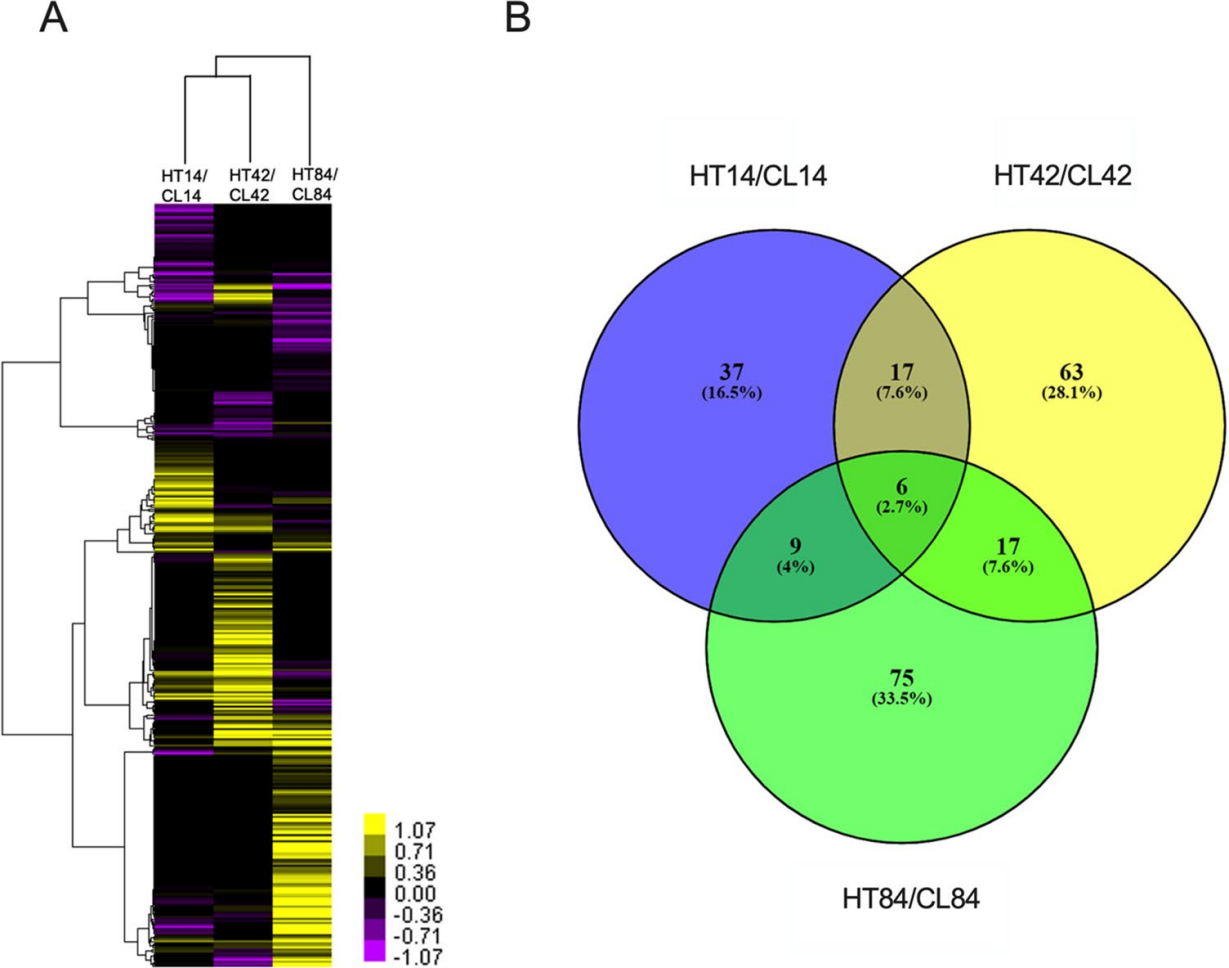
iTRAQ phosphoproteomics analysis of the mammary gland of dairy cows at three time points during lactation (14, 42, and 84 DIM). Cows were either cooled (CL, *n* = 4, access to fans and water soakers) or heat stressed (HT, *n* = 4, no access to cooling devices) during the dry period (approximately 46 days pre-calving) and mammary tissue collected in their subsequent lactation, when all cows were managed as a single group and cooled. (**A**) Heat map displays unsupervised hierarchical clustering of phosphoproteins. Bars represent fold changes in phosphoprotein abundance between CL and HT at each time point. Yellow and purple colors represent up- and down-regulated phosphoproteins in HT relative to CL, respectively (**B**) Venn diagram showing overlap of the number of differentially expressed phosphoproteins identified by iTRAQ proteomics. Phosphoproteins were considered differentially expressed between treatments at *P*-value < 0.05 and fold change > 1.3 or < 0.7.

During lactation, across all time points and treatments, a total 1237 phosphorylated proteins were identified and annotated in the bovine mammary gland (Supplementary Table S1). Among them, 890 of 1237 proteins were identified from proteomics work above, but an additional 347 proteins were identified from the phosphoproteomics work only. 1108 phosphoproteins were categorized using IPA into cellular locations and functions. Similar to the total proteins, phosphoproteins were largely located in the cytoplasm (*n* = 647) and function as enzymes (*n* = 267) (Supplementary Fig. S3). We identified 224 differentially abundant phosphoproteins between CL and HT treatments, across the three time points. More phosphoproteins had greater abundance than lower abundance in the mammary gland of HT cows across all three time points (Fig. 3A). More differentially abundant phosphoproteins were identified at 42 and 84 DIM relative to 14 DIM. The following six phosphoproteins were differentially abundant at all three time points (Fig. 3B); cationic trypsin (TRY1), heat shock 27 kDa protein 1 (HSPB1), membrane-associated progesterone receptor component 1 (PGRMC1), prothymosin alpha (PTMA), D-dopachrome tautomerase (DDT), and arginine and glutamate-rich protein 1 (ARGLU1). TRY1 and DDT were increased in HT relative to CL at all time points. HSPB1 was increased in HT at 14 and 42 DIM and decreased in HT relative to CL at 84 DIM. PGRMC1, PTMA, and ARGLU1 were all decreased in HT at 14 and 84 DIM and increased in HT compared to CL at 42 DIM.

**Figure 4:**
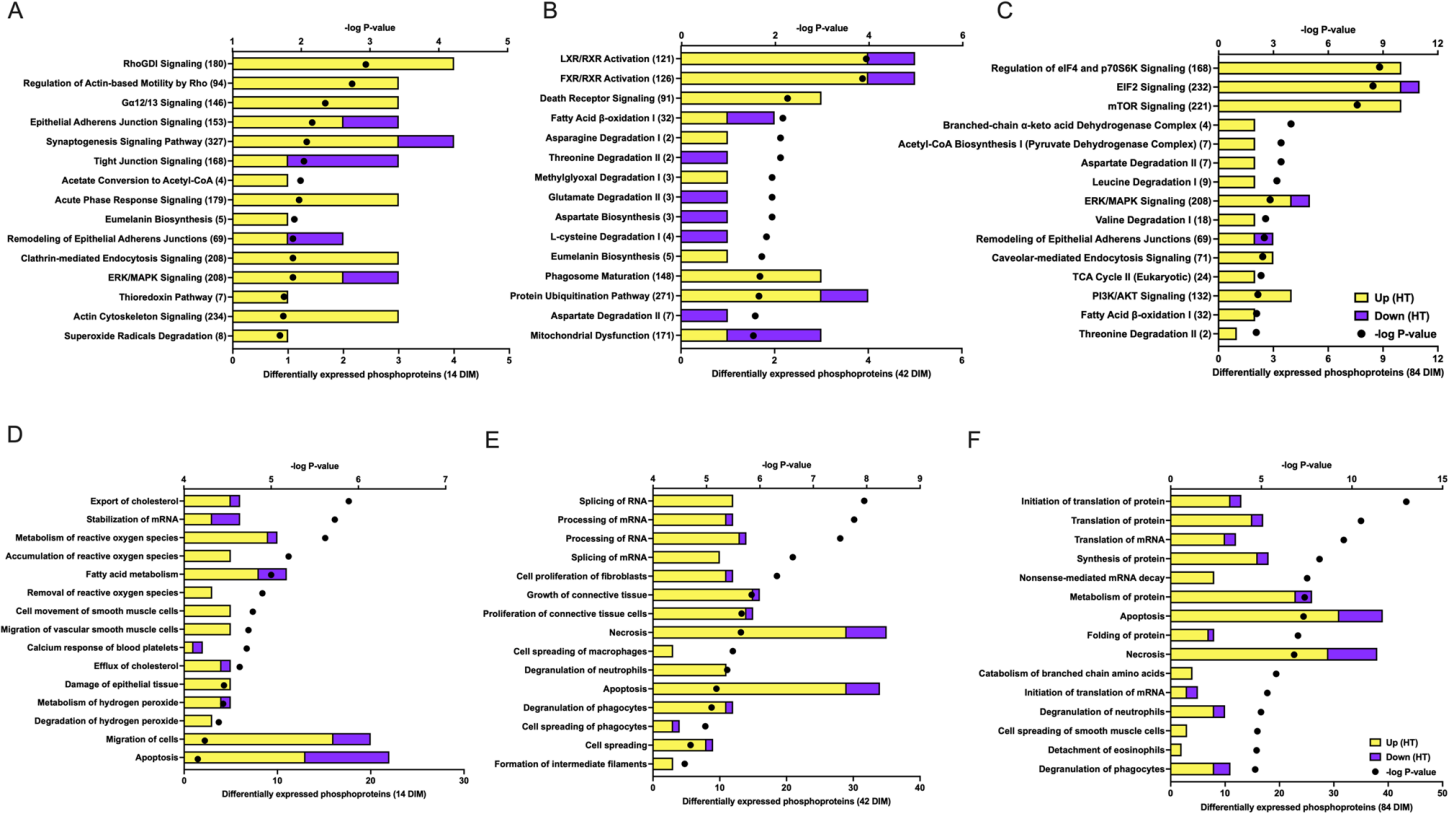
Enrichment analysis of differentially expressed phosphoproteins showing the top 15 pathways (**A**–**C**) and biological functions (**D**–**E**) in the lactating bovine mammary gland. Cows were either cooled (CL, *n* = 4, access to fans and water soakers) or heat stressed (HT, *n* = 4, no access to cooling devices) during the dry period (approximately 46 days pre-calving). Mammary tissue was collected in the subsequent lactation at 14, 42, and 84 DIM, when all cows were managed as a single group and cooled. Enrichment analysis was performed by Qiagen Ingenuity Pathway Analysis (IPA, QIAGEN Inc., https://www.qiagenbioinformatics.com). Numbers in parentheses indicate the total number of molecules in the pathway in the IPA database. Upregulation or downregulation refers to phosphoprotein expression in HT relative to CL cows.

Top pathways and functions of phosphoproteins affected by dry period heat stress at 14 DIM were related to redox balance (e.g. *thioredoxin pathway, superoxide radicals degradation, metabolism, accumulation, and removal of reactive oxygen species*), cell organization and structure (e.g. *epithelial adherens junctions signaling, tight junction signaling, remodeling of epithelial adherens junctions, RhoGDI signaling*), and lipid metabolism (Fig. 4A,D, Supplementary Table S2). Differentially abundant phosphoproteins at 84 DIM enriched in redox pathways were largely more abundant in HT relative to CL cows, including thioredoxin (TXN) and Cu–Zn superoxide dismutase (SOD1). Actin-related protein 2/3 complex subunit 5 (ARPC5) and myosin light polypeptide 6 (MYL6) were two phosphorylated proteins in the cell structure pathways that were more abundant in HT than in CL whereas catenin α-1 (CTNNA1) was less abundant in the phosphorylated form in HT cows. Differentially phosphorylated proteins with functions in fatty acid and cholesterol metabolism included proteins such as, acetyl-CoA synthetase long chain family member 1 (ACSL1), apolipoprotein A-IV (APOA4), and annexin A6 (ANXA6), which were all more abundant in HT cows.

At 42 DIM, phosphoproteins involved in fatty acid and amino acid metabolism, transcription and translation, and cell structure and survival (e.g. *formation of filaments, growth, and proliferation of connective tissue, apoptosis*) were differentially abundant between HT and CL (Fig. 4B,E, Supplementary Table S2). Except for serum albumin (ALB), all differentially abundant phosphoproteins at 42 DIM with functions in transcription and translation were more abundant in the phosphorylated form in HT compared to CL cows. In the cell structure and survival pathways, proteins were roughly equal in abundance of phosphorylation between the two treatment groups. For example, in the apoptosis pathway, MICOS complex subunit MIC60 (IMMT) was less phosphorylated whereas cytochrome b5 (CYB5A) was more phosphorylated in HT than CL.

Top pathways and functions affected by differentially abundant phosphoproteins at 84 DIM were fatty acid and amino acid metabolism, translation, and tissue necrosis (Fig. 4C,F, Supplementary Table S2). Proteins in fatty acid and amino acid metabolism pathways were more phosphorylated in HT relative to CL (e.g. GOT2, IVD). Differentially phosphorylated proteins in translation pathways were similar to the differentially abundant proteins, including many ribosomal proteins. The majority of the differentially phosphorylated proteins in the translation pathway at 84 DIM were more phosphorylated in HT than CL cows. Common affected pathways and functions across time points included cell structure and organization, translation, cell death, and macronutrient metabolism.

### Comparison of differentially expressed proteins and phosphoproteins

**Figure 5:**
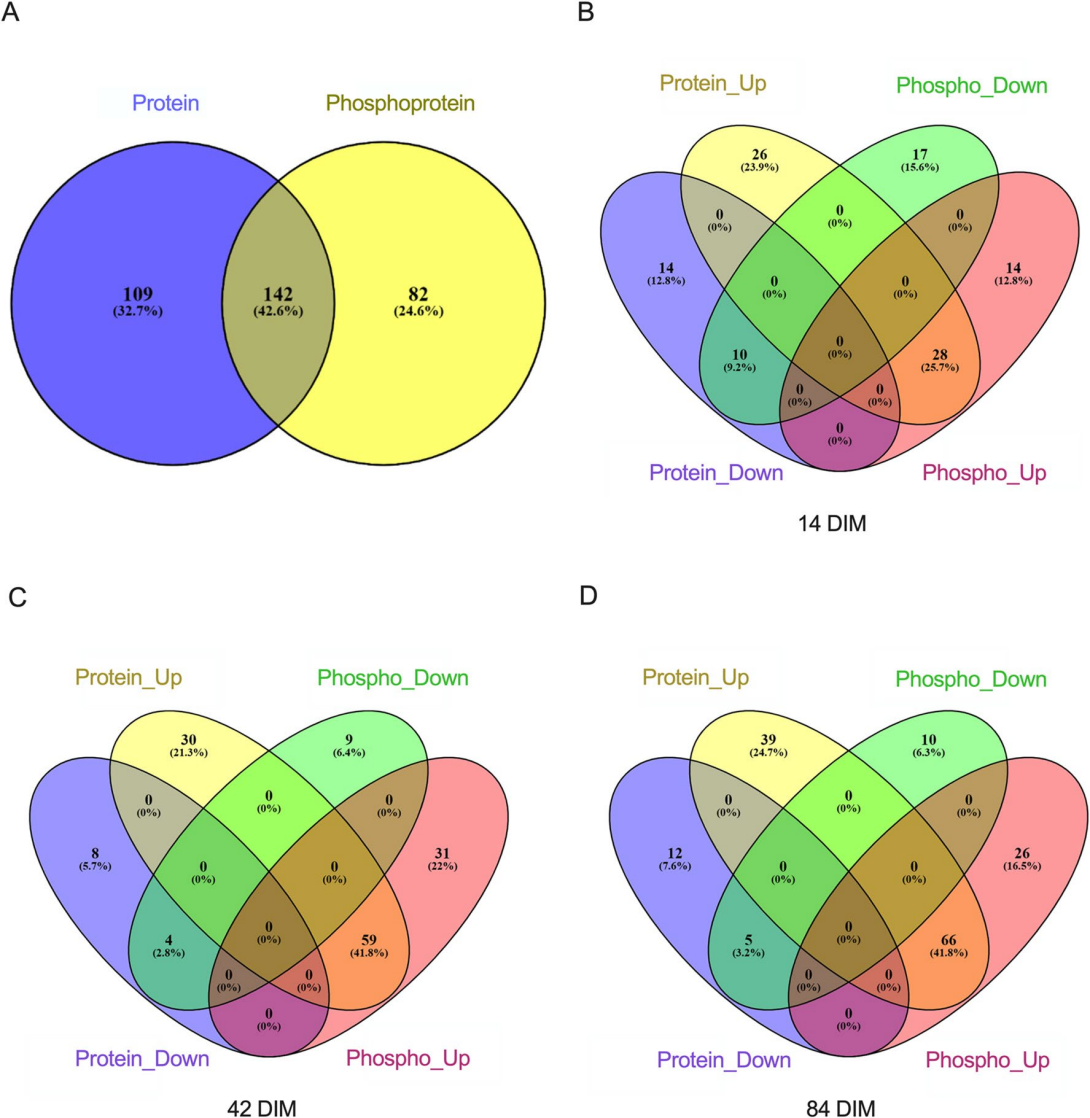
Venn diagrams displaying the overlap between the number of differentially expressed proteins and phosphoproteins (**A**) across all time points and at (**B**) 14, (**C**) 42, and (**D**) 84 DIM. Numbers of proteins or phosphoproteins are displayed along with the percent relative to total proteins or phosphoproteins, respectively. Cows were either cooled (CL, *n* = 4, access to fans and water soakers) or heat stressed (HT, *n* = 4, no access to cooling devices) during the dry period (approximately 46 days pre-calving) and mammary tissue was collected in their subsequent lactation at 14, 42, and 84 DIM, when all cows were managed as a single group and cooled. Proteins and phosphoproteins were considered differentially expressed between treatments at *P*-value < 0.05 and fold change > 1.3 or < 0.7. Protein_Up refers to upregulated proteins, Protein_Down is downregulated proteins, Phospho_Up refers to greater protein phosphorylation, and Phospho_Down indicates lower protein phosphorylation in the HT group relative to CL.

Most of the 224 differentially abundant phosphoproteins derived from differences between HT and CL in protein abundance and thus enriched pathways and functions for proteins and phosphoproteins were relatively congruent. Out of the 224 phosphoproteins, 82 exhibited differential phosphorylation (i.e., higher or lower phosphoprotein abundance) due to dry period heat stress, with no detectable difference in total protein abundance between HT and CL cows (Fig. 5A). In all cases where a differentially abundant protein was also differentially phosphorylated between groups, the direction of fold change difference between groups was in the same direction (i.e., total protein-UP/phosphoprotein-UP or total protein-DOWN/phosphoprotein-DOWN). At 14 DIM, 28 proteins were more abundant and were more phosphorylated whereas 10 proteins were less abundant and less phosphorylated in HT compared to CL cows (Fig. 5B). Fifty-nine proteins were more abundant, and their phosphorylation was greater in HT relative to CL, at 42 DIM and only four were lower in abundance and less phosphorylated in HT relative to CL (Fig. 5C). At 84 DIM, 66 proteins were more abundant and more phosphorylated whereas five proteins were less abundant and less phosphorylated in HT versus CL cows (Fig. 5D).

### Validation of protein expression

Protein expression derived from the iTRAQ technology was validated using targeted analysis (a selected ion monitoring method). 157 peptides from ten proteins were included to identify and quantify protein expression. Protein expression from targeted analysis were largely congruent with iTRAQ expression data (Supplementary Table S3).

### Network analysis of the proteome and phosphoproteome

STRING network analysis revealed 13 clusters for differentially abundant proteins between HT and CL cows at 14 DIM (Supplementary Fig. S4A). The two main enriched pathways at this time point were mRNA processing and complement and coagulation cascades containing 7 and 4 differentially abundant proteins, respectively. At 42 DIM, 12 interacting proteins were enriched in the RNA splicing pathway (Supplementary Fig. S4B). The largest number of protein clusters occurred at 84 DIM where the top 3 enriched categories were translation and mRNA processing (Supplementary Fig. S4C). Furthermore, at this time point, a cluster containing 9 differentially abundant proteins was enriched in pathways of proton transmembrane transport. Network analysis for differentially abundant phosphoproteins were similar to proteins. The top enriched cluster for each time point was related to transcription and translation (Supplementary Fig. S5). Additionally, at 84 DIM, 7 of the phosphoproteins altered by heat stress were clustered in pathways related to oxidative phosphorylation.

## Discussion

Adverse effects of heat stress during the prepartum dry period persist into the subsequent lactation even when the stressor is removed, and cows are exposed to the same environment and cooled postpartum^7–11^. However, regulation of these negative outcomes at the translational and post-translational levels has not yet been elucidated. In the present study, we revealed that the expression of more than 200 proteins in the lactating mammary gland and their phosphorylation status were altered by prior exposure to heat stress during the dry period. Overall, these proteins and phosphoproteins were largely involved in immune function, cell structure and organization, nutrient metabolism, oxidative stress, and the cellular stress response.

Among the most significant main effects of dry period heat stress on the mammary gland in early lactation (14 DIM in the present study), was cell and tissue re-organization as indicated by enriched pathways including, *remodeling of epithelial adherens junctions, RhoGDI signaling, epithelial adherens junctions signaling, migration of smooth muscle cells, organization of cytoskeleton, microtubule dynamics, tight junction signaling, organization of cellular membrane, and formation of filaments*. In congruence, disruption of epithelial junction integrity has been documented in multiple tissues, including the mammary gland, due to heat stress^26,27^. Further, in vitro and in vivo proteomics studies have also reported heat stress-induced enrichment of protein categories related to cell morphology, including epithelial junction assembly and organization^22,25^. The functional units of the mammary gland (i.e., lobuloalveolar structures) begin developing prepartum (in mid gestation) through proliferation and differentiation of cells at the terminal ends of the ductal branches^28^. The number of milk secreting cells and their secretory activity and structural organization are critical for milk production throughout the lactation period^15,29,30^. Thus, alterations in mammary parenchymal development during gestation impact milk production in the subsequent lactation^31^. Indeed, we have previously reported that relative to cooled cows, the mammary glands of cows heat stressed when dry were comprised of fewer alveoli and greater stromal connective tissue area in the next lactation, which was associated with a 3.7 kg/day reduction in milk yield across lactation^18^. Together, these studies suggest that an increase in abundance and phosphorylation of mammary gland proteins involved in tissue remodeling in HT cows contribute to architectural aberrations at the cellular and tissue levels that impair lactation performance.

Another main effect of heat stress during the dry period on the early lactating mammary gland was redox balance (e.g., *synthesis and metabolism of ROS, accumulation and removal of ROS, metabolism and degradation of hydrogen peroxide*). Although elevated ROS production appears to be rather commonplace during the transition period in high-producing dairy cattle^32,33^, our study and others indicate that oxidative stress is particularly prevalent among heat stressed cattle. Bernabucci et al.^34^ observed that cows calving in summer had greater erythrocyte antioxidant activity and lipid peroxidation, a marker of oxidative damage, during the transition period relative to cows calving in spring. Oxidative stress in HT cows may be attributed to mitochondrial dysfunction^35^, but ROS are also generated through phagocytic oxidative burst as part of the inflammatory process^36^. The results herein indicate that oxidative stress experienced by HT cows are attributed, at least in part, to a pro-inflammatory response. In the present study, C-reactive protein (CRP) was more abundant and had increased phosphorylation in HT cows relative to CL cows at 14 DIM. C-reactive protein is a positive acute phase protein that induces superoxide ion production by stimulating oxidative burst in phagocytic cells, such as macrophages and neutrophils^37^. Elevated ROS cause oxidative stress when antioxidant defense mechanisms are inadequate to neutralize the damaging radicals. In the present study, there was a higher abundance of phosphorylated superoxide dismutase 1 (SOD1) in the mammary gland of HT cows. SOD1 is a major antioxidant enzyme activated through phosphorylation and transforms superoxides into less damaging hydrogen peroxide^38^. Hydrogen peroxide is then degraded by antioxidant enzymes, such as catalase, glutathione peroxidase, and peroxiredoxins^39,40^. Although more SOD appeared to be activated (i.e., greater phosphorylation) in HT cows in our study, other antioxidants (e.g., glutathione peroxidase, catalase, peroxiredoxins) were not increased in the HT cows, suggesting heat stress may induce redox imbalance and oxidative stress.

Major pathways affected by dry period heat stress in the bovine mammary gland at peak and late lactation (i.e., 42 and 84 DIM), were related to amino acid metabolism (e.g., *asparagine degradation, threonine degradation, L-cysteine degradation, glutamate degradation, 4-hydroxyproline degradation, aspartate biosynthesis, aspartate degradation, valine degradation, leucine degradation*). In the process of milk protein biosynthesis, branched chain amino acids (BCAA) are degraded, liberating amino acids that can be used to synthesize non-essential amino acids (NEAA), such as glutamate, asparagine, aspartate, and cysteine^41^. Based on differentially abundant and phosphorylated proteins in our data set, pathways for BCAA degradation (e.g., *leucine degradation, valine degradation, catabolism of branched chain amino acids*) were upregulated in HT cows, whereas aspartate biosynthesis and glutamate, l-cysteine, and aspartate degradation pathways were downregulated. Further, alpha-S1 casein (at 14 and 42 DIM) and kappa casein (at 42 and 84 DIM) were more abundant in the HT cows, which contrasts an in vitro study where casein proteins were reduced in heat stressed bovine mammary epithelial cells directly exposed to hyperthermic conditions^22^. However, discrepancies between studies may derive from high experimental temperatures used in in vitro studies, isolation of cells in vitro, and differences in duration of heat exposure between studies. Our results appear indicative of elevated milk protein synthesis in HT cows, however, HT cows had a lower milk protein percentage compared to CL cows. Due to the lower overall yield of HT cows, total milk protein yield was also reduced in the HT relative to CL cows. In our study, aspartate aminotransferase (GOT2), a protein that is less abundant and less phosphorylated in HT cows and appears in several enriched amino acid pathways (e.g., *l-cysteine, glutamate, aspartate, and 4-hydroxyproline degradation*) and GCAT (less abundant and less phosphorylated in HT, *threonine degradation pathway*) are involved in energy production as well as amino acid metabolism. GOT2 is a key enzyme in the malate aspartate shuttle, which is a mechanism to transfer reducing equivalents across the impermeable inner mitochondrial membrane for use in oxidative phosphorylation^42^. GCAT plays a role in mitochondrial respiration through mitochondrial glycine production and its downregulation contributes to mitochondrial aging^43^. These studies suggest that lower abundance and reduced phosphorylation of these enzymes contribute to mitochondrial dysfunction, a pathway that was also enriched in our dataset. It is possible that changes in abundance and phosphorylation of these proteins had a greater effect on mitochondrial function than milk protein synthesis. However, this idea needs to be tested experimentally.

 Percent milk fat was greater in HT relative to CL cows across the lactation following exposure to dry period heat stress. However, total milk fat yield was significantly lower in HT cows due to overall lower milk production. In our dataset, multiple proteins and phosphoproteins with functions in lipid metabolism were altered due to heat stress. At 14 DIM, perilipin (PLIN), a lipid droplet associated protein that participates in lipid droplet secretion from mammary epithelial cells^44^ and acyl-CoA synthetase long chain family member 1 (ACSL), with a putative role in long chain fatty acid uptake by mammary cells^45^, were more abundant in HT cows. On the contrary, acetyl CoA carboxylase (ACACA), a rate limiting enzyme in milk fatty acid synthesis, was decreased in HT cows at 84 DIM. ACSL and acetyl CoA synthetase (ACSS 2) were more phosphorylated in HT cows at 14 DIM. Other key regulatory proteins and enzymes involved in milk lipid synthesis, such as fatty acid synthase, sterol regulatory element-binding proteins, and diacylglycerol acyltransferase were either not identified in our dataset or were not differentially abundant or differentially phosphorylated between the treatment groups at any time point. Changes in the abundance and phosphorylation status of certain proteins with lipid metabolism function may be associated with the higher milk fat concentration but lower total yield observed in HT cows.

Across all lactation time points evaluated in our study, prior exposure to heat stress during the dry period altered the phosphorylation status and abundance of proteins involved in immune function and inflammatory pathways (e.g. *leukocyte extravasation signaling, acute phase response signaling, phagosome maturation, degranulation of blood platelets, degranulation of phagocytes, cell flattening of macrophages, cell spreading of macrophages, detachment of eosinophils, diapedesis of phagocytes, degranulation of neutrophils*). The majority of proteins in these pathways (e.g., CNN2, RHOA, ITGB1, PIGR, RAB18) were increased and had increased phosphorylation in the lactating mammary gland in response to dry period heat stress. Moreover, acute phase proteins (e.g., ITIH4 and CRP) were increased and were more phosphorylated in HT cows and phosphorylation of S100A9, a Ca- and Zn-binding protein involved in inflammation^46^, was greater in HT cows. Numerous studies provide evidence of immune dysregulation and inflammatory responses in heat stressed animals^23,47–49^. In a proteomics analysis of blood samples from heat stressed, mid-lactation, Holstein cows, Min et al.^50^ found that proteins involved in both innate and adaptive immune responses were affected by heat stress, including upregulation of coagulation pathways and downregulation of the complement activation system. Similar to the present study, the phagosome maturation pathway in the post-parturient liver of cows heat stressed when dry was enriched due to protein upregulation^35^. Further, previous studies from our group using the same model of dry period heat stress as the present study reported lower neutrophil phagocytosis and oxidative burst and lower proliferation of lymphocytes in response to a mitogen during the subsequent lactation, whereas no differences were observed between HT and CL cows during the dry period when the thermal treatments were applied^8,9^. These studies indicate residual effects of dry period heat stress on innate immune function during the subsequent lactation and corroborate the proteomic and phosphoproteomic changes identified in the present study. Notably, immune alterations are also observed in calves born to HT dams, indicating that effects of in utero hyperthermia persist postnatally^51,52^. Alterations in immune responses of HT animals likely contribute to a greater risk of infection and disease, as observed in heat stressed cattle^53^.

In accordance with published in vitro and in vivo heat stress studies^19,21,24,54^, we found evidence of thermal induction of cytoprotective responses in the mammary gland of HT cows. In our study, the *apoptosis* pathway was enriched across all three lactation stages evaluated. Differentially abundant and differentially phosphorylated proteins in this pathway were largely involved in the cellular stress response and had cell survival functions. In the present study, several anti-apoptotic members of the heat shock family (e.g., HSPB1/Hsp27, ST13/Hip, and DNAJA1/Hsp40^55–57^) were increased and were more phosphorylated in HT cows. The heat shock response is induced by a number of cell stressors, including heat stress, and involves a family of molecular chaperones that serve a cytoprotective role by trapping, re-folding, or degrading misfolded proteins^58^. We also found that 14–3–3 protein zeta/delta (YWHAZ), a protein that inhibits apoptosis through a number of pathways and downstream targets, including the PI3k/Akt pathway, caspases, and cadherins^59,60^ was also more abundant and more phosphorylated in HT cows. These results suggest long-term upregulation of cytoprotective proteins that effectively prevent the persistence of excessive cell apoptosis beyond the initial thermal insult. Indeed, immunohistochemical analysis showed similar proportions of mammary epithelial and stromal cells undergoing apoptosis across lactation in cows that were exposed to heat stress or cooling conditions during the dry period^10,18^.

Although the majority of proteins in the present dataset were both more abundant and more phosphorylated or both less abundant and less phosphorylated, 109 proteins had changes in abundance between treatment groups with no difference in phosphorylation status. For example, fibromodulin (FMOD) was increased in HT at 42 DIM, but not differentially phosphorylated. Protein phosphorylation is a ubiquitous covalent protein modification, whereby protein kinases catalyze the reversible addition of a phosphate group from ATP or GTP to the side chain of specific amino acids residues, typically serine, threonine, or tyrosine^61,62^. Phosphorylation regulates protein function, stability, localization, and protein–protein interactions^63,64^. However, many other post-translational modifications occur, such as ubiquitination, glycosylation, acetylation, methylation, and others, which can alternatively control protein activity^65^. Indeed, FMOD undergoes glycosylation^66^ and can be modulated through sulfation^67^. Thus, for some proteins, total protein abundance may not correlate with phosphoprotein abundance.

In addition, there were 82 proteins with differential phosphorylation status but similar total protein abundance between HT and CL cows. For example, APOA4 was more phosphorylated in HT and DDX17 was less phosphorylated in HT relative to CL with no difference in total protein abundance between groups. Changes in phosphorylation status without changes in protein abundance have been documented in a number of studies comparing the proteomes and phosphoproteomes of diverse organisms and cell types and is indicative of protein modulation at the post-translational rather than the transcriptional level^68–71^.

Network analysis revealed common functional clusters of interacting proteins and phosphoproteins across the lactation period, altered by dry period heat stress. Across time points, clusters containing the most differentially abundant proteins were functionally associated with the processes of transcription and translation, especially RNA splicing and mRNA processing, which may reflect the dynamic shifts in tissue activities across different stages of the lactation cycle. Using a similar experimental model as the present study, differentially abundant proteins involved in transcription and translation were also identified in the liver of early lactation cows after exposure to heat stress during the dry period^35^. In that same study, the top enriched pathways affected by heat stress were oxidative phosphorylation and mitochondrial dysfunction^35^. Similarly, in the present study, differentially abundant proteins and phosphoproteins between HT and CL cows clustered in networks enriched in proton transport and oxidative phosphorylation. Together, these studies suggest heat stress during the dry period has carry over effects on mitochondrial function and energy metabolism across tissue types.

## Conclusions

Herein, we quantified dynamic proteomic and phosphoproteomic changes in the lactating mammary gland as a result of prior exposure to heat stress during the dry period. We identified more than 200 proteins in the lactating mammary gland that were differentially abundant and differentially phosphorylated between cows that were heat stressed compared to cows that were actively cooled during the dry period. Pathways and functions enriched by the differentially abundant proteins and differentially phosphorylated proteins were related to redox status, immune function, tissue remodeling, and nutrient metabolism. Changes in the abundance or phosphorylation of these proteins in the mammary gland might result in alterations of key pathways that may contribute to previously reported aberrations in mammary ultrastructure, oxidative stress, and immune dysregulation in thermally stressed cows, that ultimately impair milk production. Although the underlying mechanisms orchestrating these long-lasting changes in protein and phosphoprotein expression profiles was not the goal of this study, aberrant methylation patterns of ribosomal protein machinery or protein kinases, as reported previously by our group^72^ may contribute to persistent alterations in protein abundance and phosphorylation in heat stressed animals. Further investigation is warranted to elucidate regulatory mechanisms driving long-term proteomic and phosphoproteomic alterations in the mammary glands of heat stressed dry cows.

## Methods

### Animals and dry period treatment

The study was conducted at the University of Florida Dairy Unit in Hague, FL from May through October 2016. All experiments were performed in accordance with ARRIVE guidelines and were approved by and performed in accordance with guidelines and regulations of the University of Florida Institutional Animal Care and Use Committee (Protocol #201508730). Details on the experimental design, housing, and management can be found in Dado-Senn et al.^18^. Multiparous cows were dried off approximately 46 days before expected calving, then blocked by mature equivalent milk yield in the previous lactation, lactation number, and body mass at dry off and randomly assigned to one of two treatment groups, heat stressed (HT,* n* = 12) or cooled (CL, *n* = 12) until calving. Cows from each group were housed on opposite sides of the same shaded, sand-bedded, free-stall barn. Cooled cows had access to fans and water sprayers above the feed bunks. Fans ran continuously and water soakers were automatically activated for 1.5 min every 5 min when ambient temperature exceeded 21.1 °C. Heat stressed cows did not have access to these cooling devices. The Temperature-Humidity Index of the two sides of the barn (calculated as in Dikmen et al.^73^) was similar (HT 74.6 vs CL 74.4 ± 0.3) for the entire dry period^18^. CL cows had lower respiration rates and rectal temperatures relative to HT cows, confirming the success of the treatments^18^. During the dry period, cows were fed a common close-up total mixed ration to meet National Research Council recommendations. After calving, all cows were moved to an adjacent shaded, sand-bedded, free-stall barn and provided with water soakers and fans. Thus, the disparate thermal treatments occurred for approximately 46 days in the dry period only. Lactating cows were fed a total mixed ration and milked 4 times per day until 21 DIM and thereafter 2 times per day according to Dairy Unit standard operating procedures. Daily dry matter intake was similar between HT and CL cows during the dry period and lactation (recorded from calving to 42 DIM^18^). Daily milk yield and milk composition were obtained from the Dairy Unit Management Software (Afimilk Ltd., Kibbutz, Afikim, Israel).

### Mammary biopsies and protein extraction

Serial mammary biopsies were collected from a subset of six cows per treatment at 14, 42, and 84 ± 1 DIM following the Farr method^74^ with modifications by our group^10^. The biopsied tissue was rinsed with PBS, trimmed of visible fat, and snap-frozen in liquid nitrogen. Tissue was stored at − 80 °C until protein extraction. Out of the six biopsied cows, two HT and one CL cow were diagnosed with mastitis during lactation and were removed from the study. An additional CL cow was removed, at random, to maintain an equal group size of four cows per treatment group for downstream proteomic and phosphoproteomic analyses. These cows did not have any known diseases or metabolic disorders.

Approximately 50 mg of mammary tissue was homogenized in Tris-buffered phenol and extraction media containing 0.1 M Tris–HCl, 10 mM EDTA, 1.2 M sucrose, 1.2% β-mercaptoethanol using a liquid nitrogen-cooled mortar and pestle. The homogenate was then incubated at 15 °C and 1200 rpm for 1 h, followed by centrifugation at 15 °C for 30 min at 7000×*g*. The phenol and protein phases were transferred to a clean microcentrifuge tube. Precipitation of phenol-extracted proteins was accomplished by adding 5 volumes of cold ammonium acetate (0.1 M) in 100% methanol and incubating at − 20 °C overnight and then collecting the precipitate by centrifugation at 4 °C for 20 min at 20,000×*g*. The pellet was subsequently washed twice with 0.1 M ammonium acetate in 100% methanol and twice with cold 80% acetone. The final pellet was resuspended with 4 M urea buffer. Protein concentration was quantified with the EZQ kit (#R33200, Molecular Probes/ Invitrogen, Eugene, OR, USA). The remaining protein was stored at − 80 °C until proteomic and phosphoproteomic analyses.

### iTRAQ-based proteomics

Proteins were quantified as previously described^75^, and dissolved in denaturant buffer [0.1% SDS (w/v)] and dissolution buffer (0.05 M triethylammonium bicarbonate, pH 8.5) in the iTRAQ Reagents 8-plex kit (#4381662, AB Sciex Inc., Foster City, CA, USA). For each sample, a total of 100 μg of protein were reduced, alkylated, trypsin-digested, and labeled according to the manufacturer’s instructions. Samples from CL cows at 14, 42, and 84 DIM were labeled with iTRAQ tags 113, 114, 115, and 119, and its corresponding experimental samples from HT cows were labeled with iTRAQ tags 116, 117, 118, and 121. Biological tetraplicates were exploited in this study and the fourth replicate was employed for the label swapping across the set (Supplementary Fig. S1).

### ITRAQ LC–MS/MS analysis

Labeled peptides were desalted with C18-solid phase extraction and dissolved in strong cation exchange (SCX) solvent A (25% (v/v) acetonitrile, 10 mM ammonium formate, and 0.1% (v/v) formic acid, pH 2.8). The peptides were fractionated using an Agilent high-performance liquid chromatographer (HPLC) 1260 with a polysulfethyl A column (2.1 Å ~ 100 mm^2^, 5 μm, 300 Å; PolyLC, Columbia, MD). Peptides were eluted with a linear gradient of 0−20% solvent B (25% (v/v) acetonitrile and 500 mM ammonium formate, pH 6.8) over 50 min followed by ramping up to 100% solvent B in 5 min. The absorbance at 280 nm was monitored, and a total of 16 fractions were collected. The fractions were desalted using ZipTip pipette tips (#ZTC18S, MilliporeSigma, Burlington, MA, USA), lyophilized, and resuspended in LC solvent A (0.1% formic acid in 99.9% water (v/v)). A Q-Exactive Plus hybrid Quadrupole-Orbitrap mass spectrometry (MS) system (Thermo Fisher Scientific, Bremen, Germany) was used with high energy collision dissociation (HCD) in each MS and MS/MS cycle. The MS system interfaced with an automated Easy nLC-1200 system (Thermo Fisher Scientific, Bremen, Germany). Each sample fraction was loaded onto an Acclaim Pepmap 100 pre-column (20 mm × 75 μm; 3 μm-C18) and separated on a PepMap RSLC analytical column (250 mm × 75 μm; 2 μm-C18) at a flow rate of 350 nl/min during a linear gradient from solvent A (0.1% formic acid (v/v) and 99.9% water (v/v)) to 25% solvent B (0.1% formic acid (v/v), 19.9% water (v/v), and 80.0% acetonitrile (v/v)) for 80 min, and to 100% solvent B for an additional 15 min. Full MS scans were acquired in the Orbitrap mass analyzer over m/z 400–2000 range with resolution 70,000 at 200 m/z. The top ten most intense peaks with charge state ≥ 2 were fragmented in the HCD collision cell with normalized collision energy of 28%, (the isolation window was 2 m/z). The maximum ion injection times for the survey scan and the MS/MS scans were 250 ms, respectively, and the ion target values were set to 3e6 and 1e6, respectively. Selected sequenced ions were dynamically excluded for 60 s.

### Enrichment of phosphopeptides

Phosphopeptide enrichment is performed using titanium dioxide and zirconium dioxide Nutip (Glygen Inc., Columbia, MD, USA). First, Nutip was equilibrated by washing the resin 10 times with 5 μl of binding solution (80% acetonitrile (v/v), 5% TFA (v/v), pH 3), and the tryptic digests were mixed at a 1:1 ratio with binding solution. The mixture was loaded onto Nutip by taking 2.5 μl aliquots and aspirating and expelling each aliquot 50 times over 30 min. Washes consisting of 80% acetonitrile (v/v), 1% TFA (v/v) (pH 3) 10 times followed. The bound phosphopeptides were eluted with 10 μl of elution solution (2% NH_4_OH (v/v), pH 11). To prevent loss of the phosphate moieties under highly basic conditions, the eluent was acidified with 2 μl of 10% formic acid (v/v) and lyophilized. The sample was then resuspended in LC solvent A and injected into the Q Exactive Plus (see above).

### Targeted LC–MS/MS analysis based on data dependent decision tree acquisition

An Orbitrap Fusion Tribrid Mass Spectrometer system (Thermo Fisher Scientific, San Jose, CA, USA) was used with collision ion dissociation (CID) in each MS and MS/MS cycle. The MS system was interfaced with an ultra-performance Easy-nLC 1200 system (Thermo Fisher Scientific, Bremen, Germany). A total of 2 μg of each sample was loaded onto an Acclaim Pepmap 100 pre-column (20 mm × 75 μm; 3 μm-C18) and then separated on a PepMap RSLC analytical column (500 mm × 75 μm; 2 μm-C18) at a flow rate of 250 nl/min of solvent A (0.1% formic acid (v/v) and 99.9% water (v/v)), followed by a linear increase from 2 to 35% solvent B (0.1% formic acid (v/v), 19.9% water (v/v), and 80% acetonitrile (v/v)) in 160 min and from 35 to 80% solvent B in 5 min, then ramping up to 98% solvent B in 1 min, and then held for 14 min. The mass spectrometer was operated in MS/MS mode scanning from 350 to 2000 m/z. The maximum ion injection times for the survey scan and the MS/MS scans were 35 ms. MS1 spectra were recorded at resolution at 120,000 FWHM from 350 to 2000 m/*z* with quadrupole isolation was followed by one MS/MS scans of the most intense precursor ions in the linear ion trap. The automated gain control (AGC) target was set to 2 × 10^5^, with a max. injection time of 50 ms. The quadrupole was used for precursor isolation with an isolation window of 1.3 m/*z*. Only precursors with charge states 2–5 with an intensity higher than 1 × 10^4^ were selected for fragmentation. The monoisotopic precursor selection (MIPS) filter was activated. The option to inject ions for all available parallelizable time was selected. Targeted MS2 spectra with different fragmentation parameters were acquired (Supplementary Table S4) and were performed in the ion trap with CID fragmentation (Rapid; NCE 35%; maximum injection time 35 ms; AGC 1 × 10^4^). The normalized collision energy (NCE) was set to 35% for each fragmentation method and one microscan was acquired for each spectrum.

### ITRAQ proteomics data analysis

The raw MS/MS data files were processed by a thorough database searching approach considering biological modification and amino acid substitution against uniprot *Bos taurus* database (45,234 entries; downloaded on March. 9th, 2018) using the ProteinPilot v4.5 with the Fraglet and Taglet searches under the Paragon™ algorithm^76^. The following parameters were considered for all the searching: fixed modification of methylmethane thiosulfonate-labeled cysteine, fixed iTRAQ modification of amine groups in the N-terminus, lysine, and phosphorylated modification in tyrosine, serine, and threonine. For protein quantification, only MS/MS spectra that were unique to a particular protein and where the sum of the signal-to-noise ratios for all the peak pairs > 9 were used for quantification. Confidence levels of protein identification were set to 95% to establish a list of proteins with a 5% false discovery rate (FDR)^77^. Relative quantification of proteins detected by unique peptides was conducted using ratios from tandem mass spectra.

### Targeted proteomics data analysis

All MS/MS samples were processed by a thorough database search considering biological modification and amino acid substitution against the database (see above) with decoy option using MASCOT 2.7.01 (Matrix Science Inc., Boston, MA, USA) under Proteome Discoverer version 2.4 (Thermo Fisher Scientific.) with the following parameters: peptide tolerance at 10 ppm, tandem MS tolerance at ± 1.00 Da, peptide charge from 2 + to 6 +, trypsin as the enzyme, Carbamidomethyl (C) as fixed modifications, and oxidation (M) and phosphorylation (S, T, Y) as variable modifications. Proteome Discoverer was used to validate the Target Decoy PSM Validator node and assign false discovery rates (FDRs) under 5%. Target peptide identifications were accepted if they could be established at greater than 99.9% probability by the Peptide Prophet algorithm^78^. Proteins that contained similar peptides and could not be differentiated based on MS/MS analysis alone were grouped to satisfy the principles of parsimony. Proteins sharing significant peptide evidence were grouped into clusters. The selected ion and detected ion area for each protein was calculated by assigning a peptide from that protein with high confidence. To determine differential expression of proteins, each sample expression was compared. The *p-values* were corrected for multiple testing by the method of Storey and Tibshirani^79^, and the significance of differential expression was assessed by t-test of *p* ≤ 0.05 with fold change > 1.5 or < 0.5.

### Statistical and functional analysis

**Figure 6:**
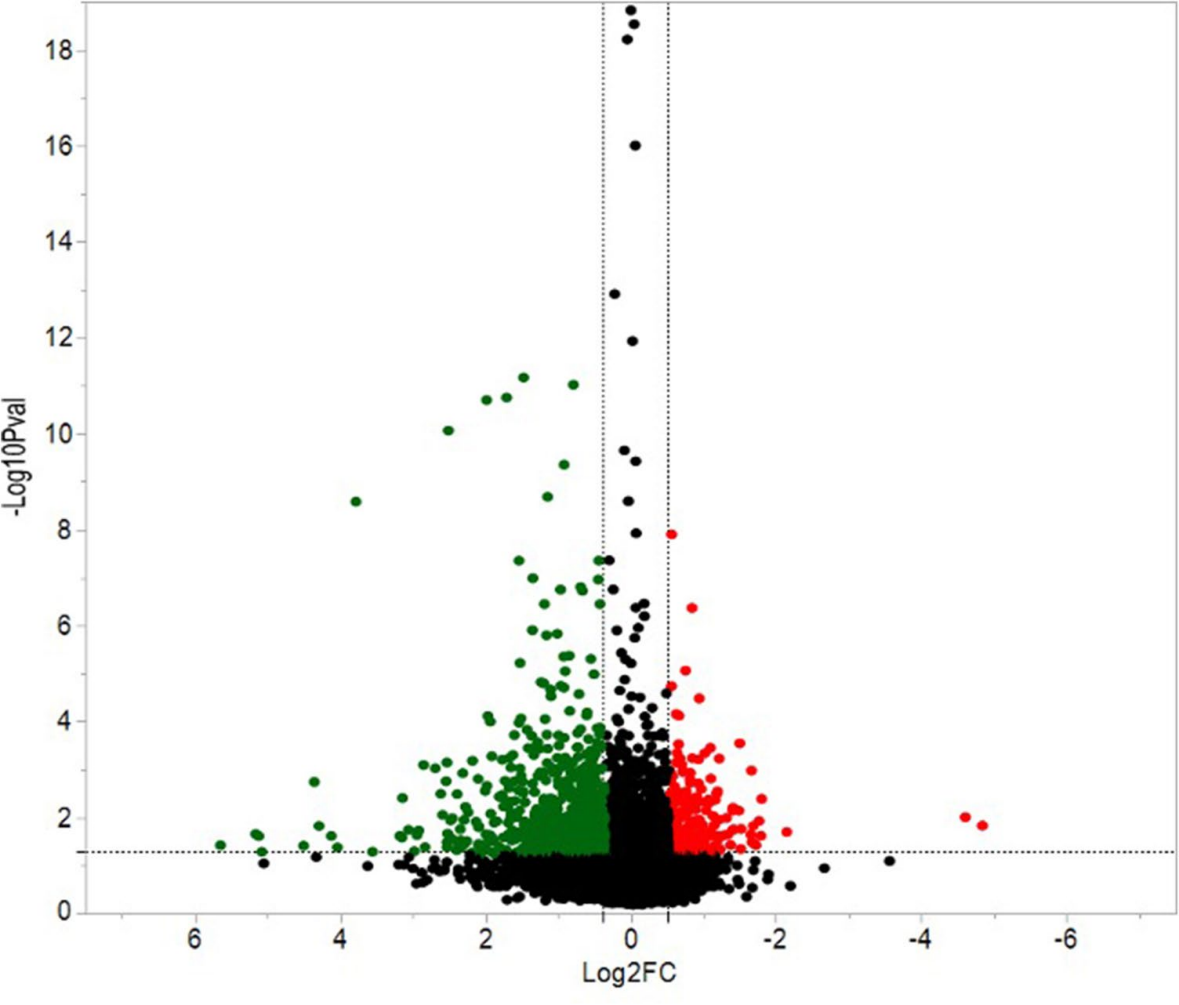
Volcano plot of proteins expressed in the mammary glands of dairy cows at three time points during lactation (14, 42, and 84 DIM). Cows were either cooled (CL, *n* = 4, access to fans and water soakers) or heat stressed (HT, *n* = 4, no access to cooling devices) during the dry period (approximately 46 days pre-calving) and mammary tissue was collected in their subsequent lactation, when all cows were managed as a single group and cooled. The x-axis is the log2 fold change for expression of the protein in HT relative to CL cows. The y-axis is the – log10 P-value for each protein. Red dots are indicative of greater protein abundance and green dots are lower protein abundance in HT relative to CL cows. Black dots represent proteins that are not significantly different in abundance between HT and CL cows.

Milk yield and components data were analyzed using PROC MIXED in SAS v9.4 (SAS Institute, Cary, NC). Models contained fixed effects of treatment (HT or CL), DIM, and an interaction between treatment and DIM. Cow ID within treatment was included as a random effect and DIM was included as a repeated measure. Milk yield and components yield were log transformed. Data are presented as back-transformed least squared means (LSM). For the identification of proteins and phosphoproteins significantly differentially expressed between HT and CL, we considered proteins that were quantified with at least three unique spectra in biological tetraplicate, along with a Fisher’s combined probability of < 0.05^80^ and a fold change < 0.7 or > 1.3, based on the volcano plot of expressed proteins (Fig. 6). Proteins and phosphoproteins differentially expressed were clustered by hierarchical clustering via complete linkage of Pearson correlations using CLUSTER 3.0, and the results were visualized by Java TreeView v. 1.1.6r4. Ingenuity Pathway Analysis (IPA, QIAGEN Inc., https://www.qiagenbioinformatics.com) was used to categorize the cellular functions and localization of all identified proteins and pathways and biological functions of the differentially expressed proteins and phosphoproteins. The MS proteomics data have been deposited to the MassIVE data depository with the data set identifier MSV000088773 (iTRAQ) and MSV000088778 (targeted proteomics) via the PRIDE partner repository^81^ with the data set identifier PXD00000. Network analysis was performed using STRING^82^ (https://string-db.org, version 11.0) to identify functional protein association networks and common functional clusters of interacting proteins and phosphoproteins across the lactation period altered by exposure to heat stress pre-calving.

### Approval for animal experiments

All animal experiments and procedures complied with ARRIVE guidelines and were approved by and performed in accordance with guidelines and regulations of the University of Florida Institutional Animal Care and Use Committee (Protocol #201508730).

## Supplementary Information

Supplementary Figures.

Supplementary Table S1.

Supplementary Table S2.

Supplementary Table S3.

Supplementary Table S4.

## Data Availability

The MS proteomics data have been deposited to the MassIVE data depository with the data set identifier MSV000088773 (iTRAQ) and MSV000088778 (targeted proteomics) via the PRIDE partner repository^81^ with the data set identifier PXD00000.
