# Supplementary material for: Carry-over effects of dry period heat stress on the mammary gland proteome and phosphoproteome in the subsequent lactation of dairy cows
Source: Sci Rep. 2022 Apr 22;12:6637. doi: 10.1038/s41598-022-10461-z (PMC9033811; doi:10.1038/s41598-022-10461-z)
Supplement: Supplementary file 1 — Supplementary Figures. [file 41598_2022_10461_MOESM1_ESM.docx]

**Carry-over effects of dry period heat stress on the mammary gland proteome and phosphoproteome in the subsequent lactation of dairy cows**

Amy L. Skibiel^1^, Jin Koh^2^, Ning Zhu^2^, Fanchao Zhu^2^, Mi-Jeong Yoo^3^, Jimena Laporta^4*^

^1^Department of Animal, Veterinary and Food Sciences, University of Idaho, Moscow, ID, 83844, USA

^2^Interdisciplinary Center for Biotechnology Research, Proteomics and Mass Spectrometry Core, University of Florida, Gainesville, FL, 32611, USA

^3^Department of Biology, Clarkson University, Potsdam, NY, 13699, USA

^4^Department of Animal and Dairy Sciences, University of Wisconsin-Madison, Madison, WI, 53715, USA

*Corresponding author:

Email: [jlaporta@wisc.edu](mailto:jlaporta@wisc.edu)

**Supplementary Figure S1.** iTRAQ sample design. Proteins were extracted from mammary tissue of lactating cows at 14, 42, and 84 days in milk. Cows were either cooled with water soakers and fans (CL, *n* = 4) or heat stressed (HT, *n* = 4, no access to cooling) for the entire dry period (~46 days). CL protein samples were labeled with iTRAQ labels 113, 114, 115, or 119, whereas HT protein samples were labeled with iTRAQ labels 116, 117, 118, or 121 using 8-PLEX iTRAQ Reagents Kits and run in 3 sets.


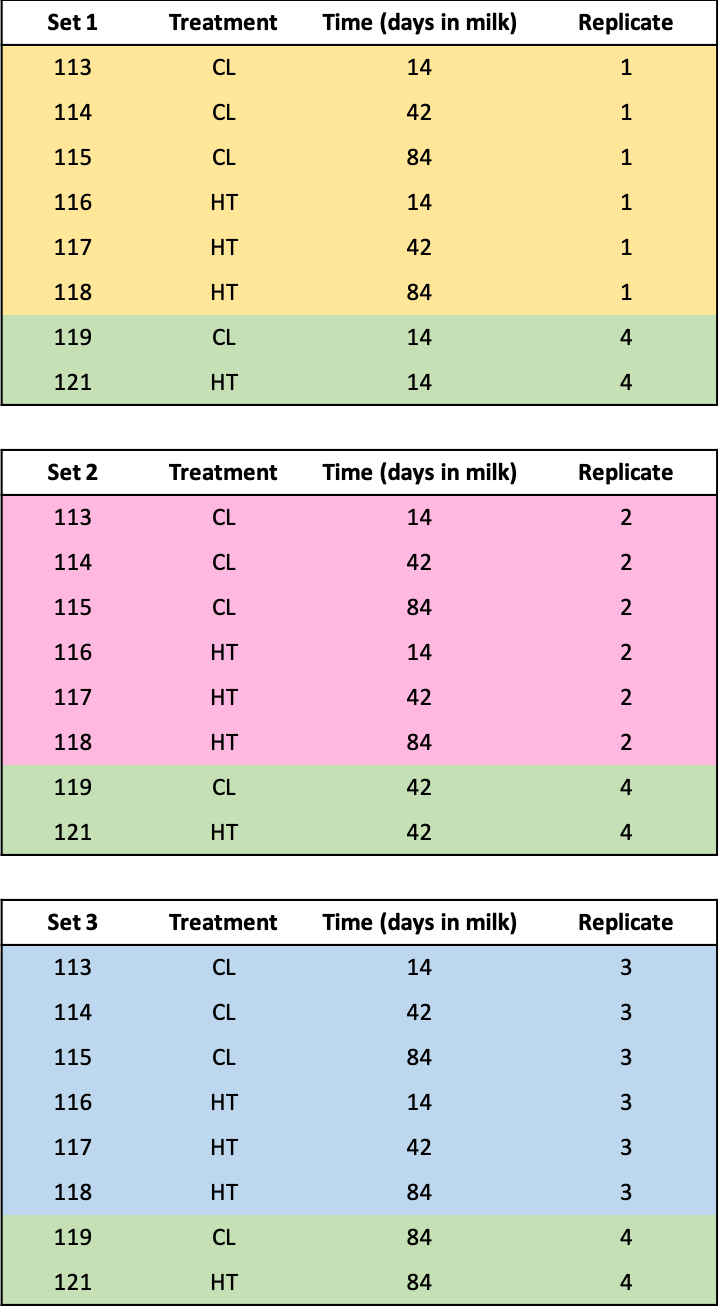


**Supplementary Figure S2.** Cellular locations (A) and functions (B) of 3,673 proteins identified in the lactating bovine mammary gland. The number of proteins is indicated in parentheses and the percent of proteins in each category is provided.

**
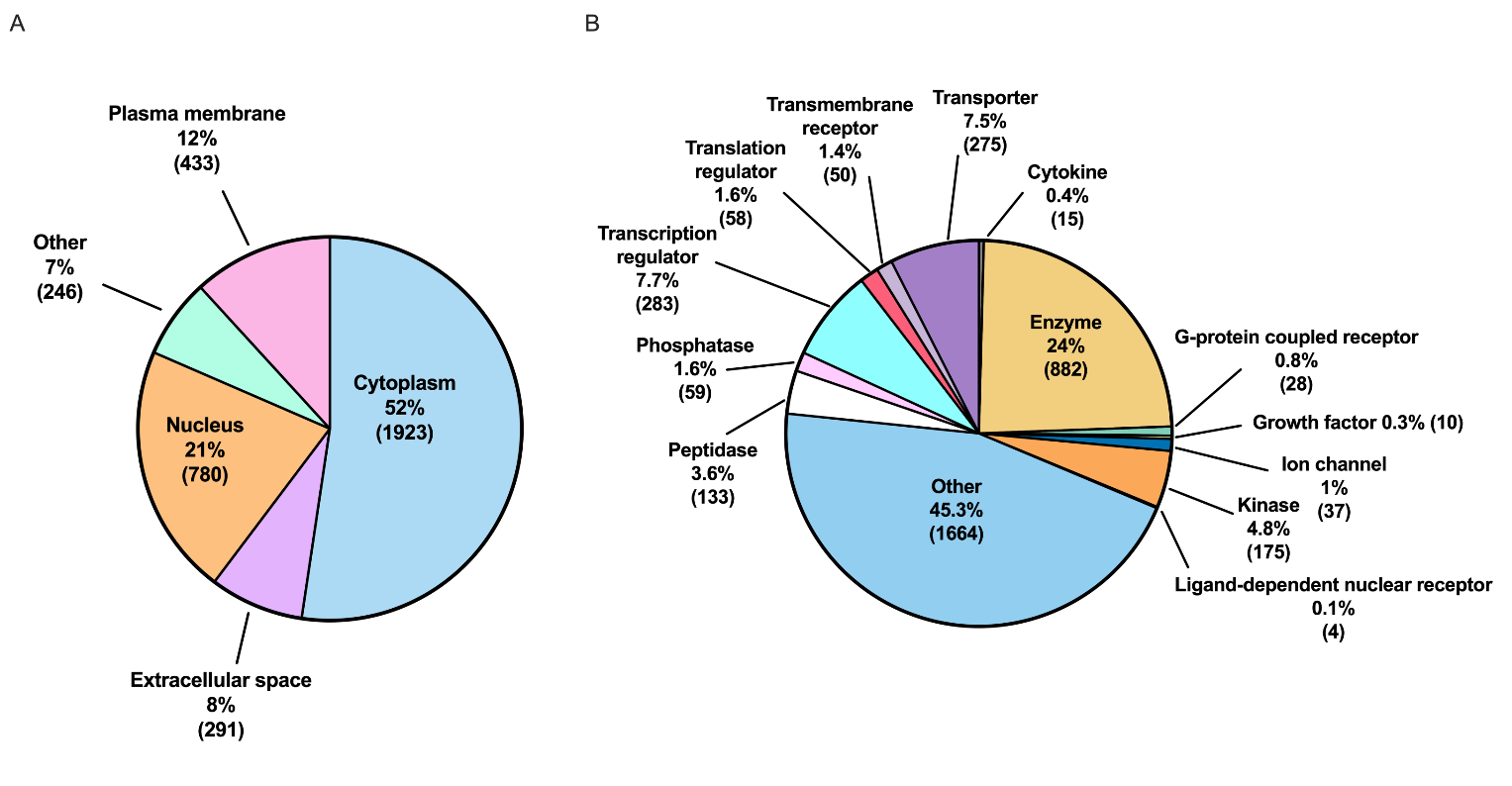
**

**
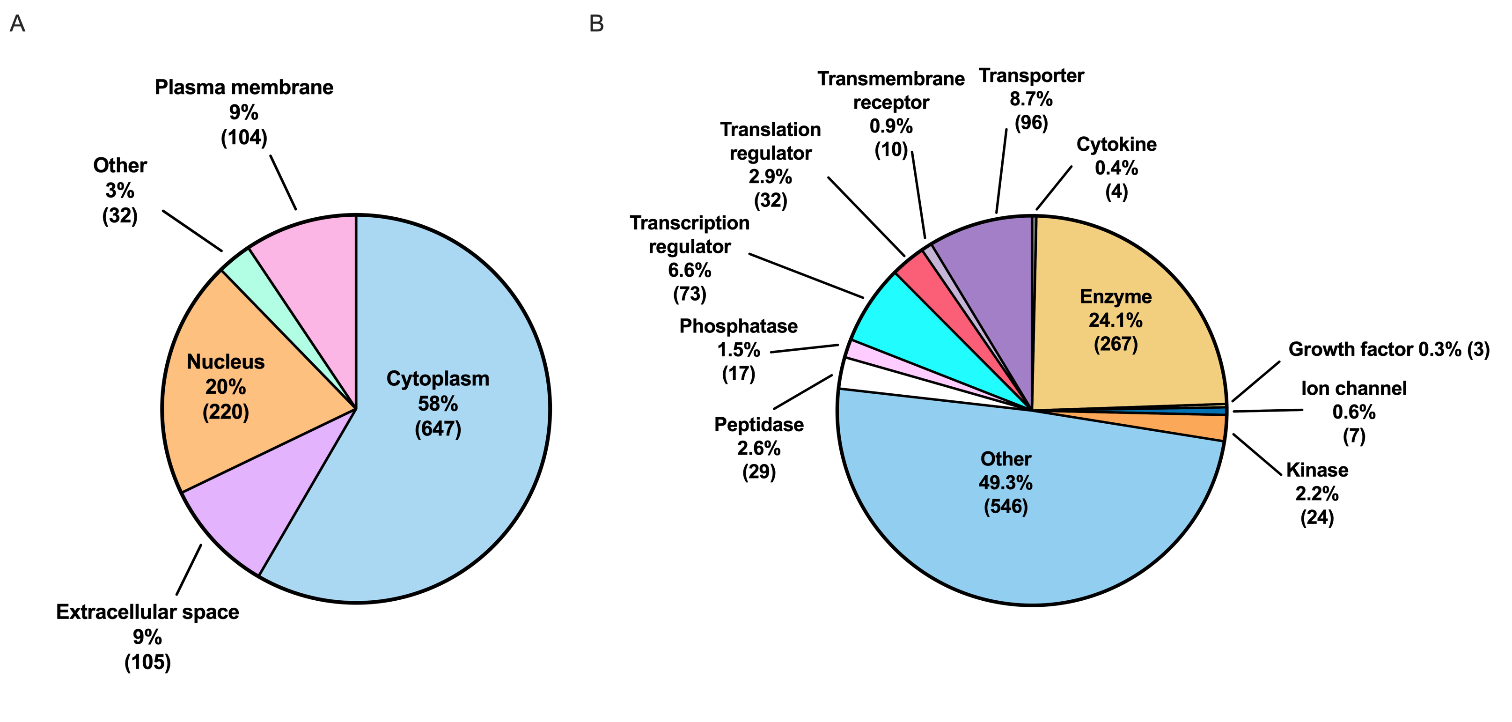
Supplementary Figure S3.** Cellular locations (A) and functions (B) of 1,108 phosphoproteins identified in the bovine mammary gland during lactation. The number of phosphoproteins is indicated in parentheses and the percent of phosphoproteins in each category is provided.

**Supplementary Figure S4.** A network of differentially expressed proteins using STRING. The red and blue halo colors indicate more or less abundant proteins in the mammary gland of HT (no access to cooling for the ~ 46-day dry period, *n* = 4) compared to CL (cooled with water soakers and fans, for the ~ 46-day dry period, *n* = 4) cows, respectively. (A) 14 days in milk (DIM), (B) 42 DIM, and (C) 84 DIM. The same color of balls indicates those proteins belong to the same cluster based on MCL clustering with a default value in STRING. Enriched biological processes or pathways are shown in the text. The thickness of the edges reflects the strength of connection between the proteins based on known interactions (either inferred from curated databases or experimental results), predicted interactions (gene neighborhood, gene fusion, and gene co-occurrence), and others (text-mining information, co-expression, and protein homology) at medium confidence score. Refer to Supplementary Table S1 for protein ID and its description. Figures were created using STRING (<https://string-db.org>, version 11.0).


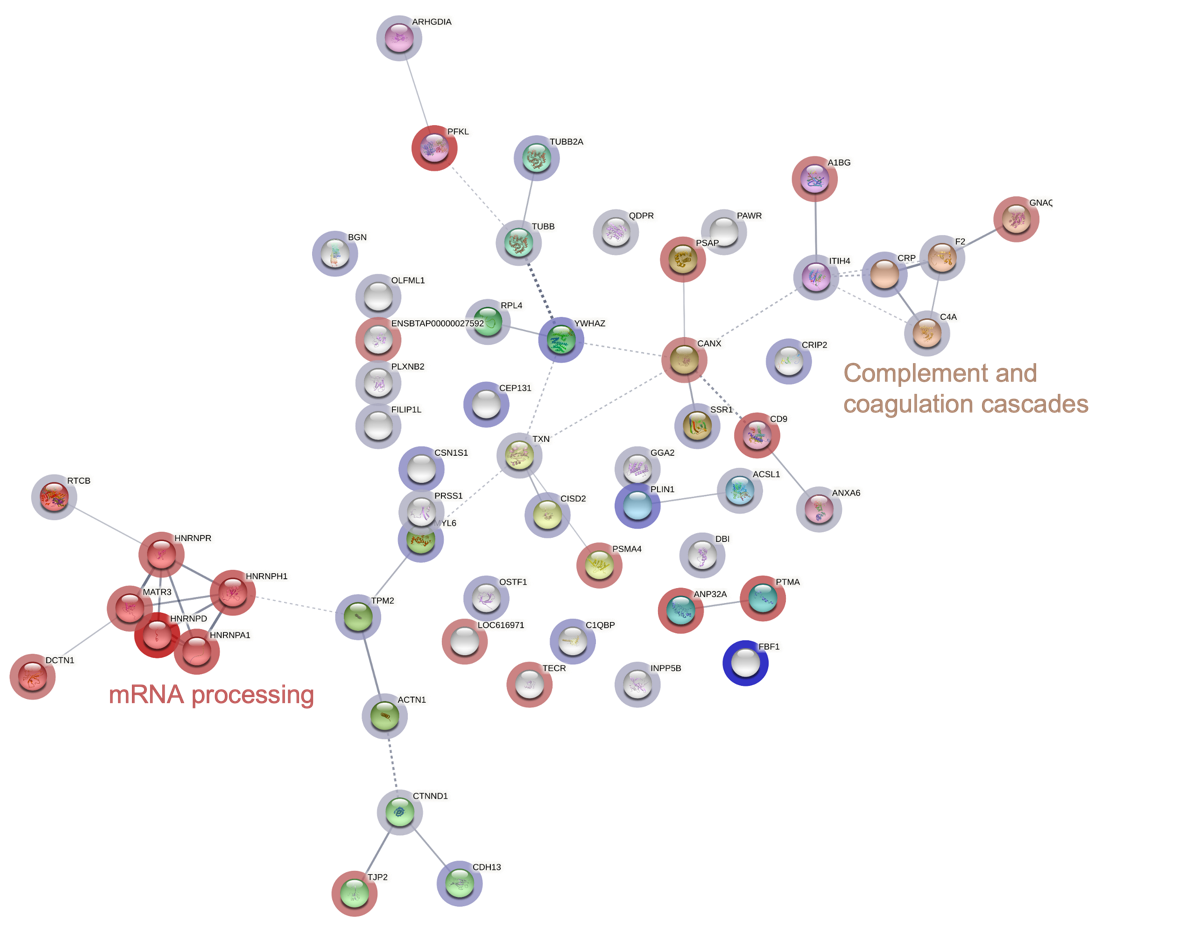


A


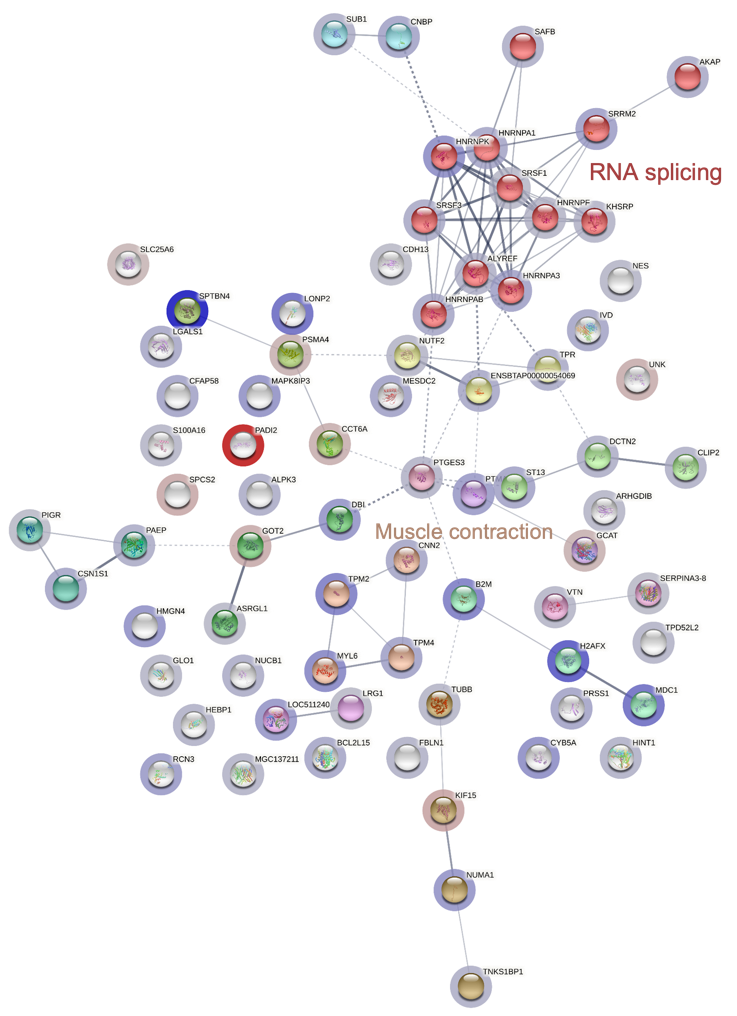
B


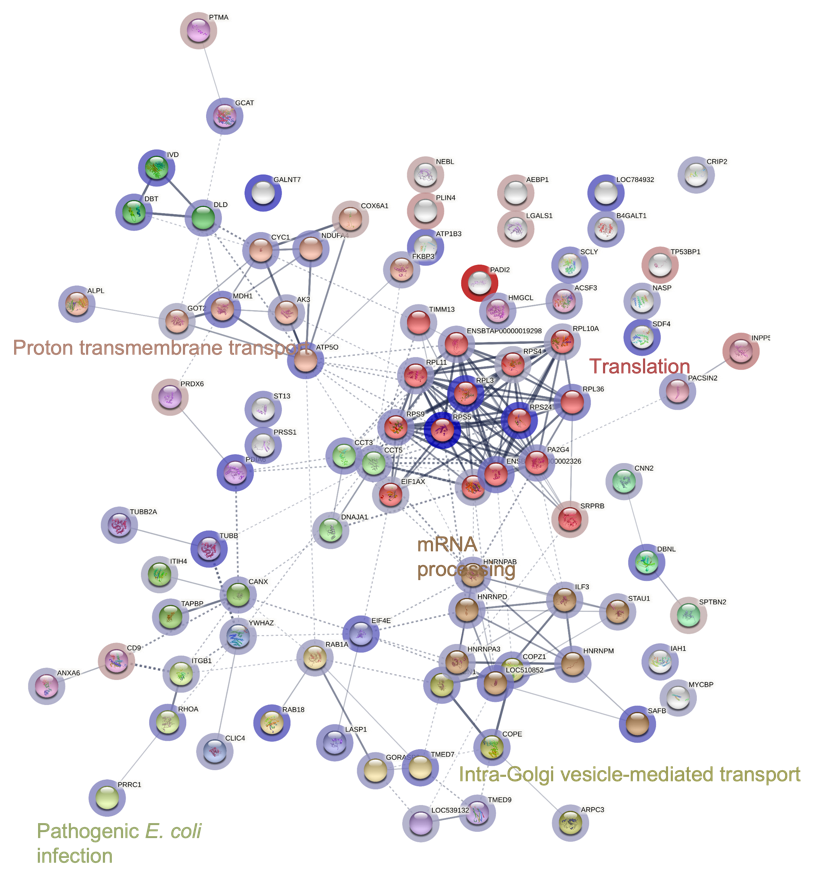
C

**Supplementary Figure S5.** A network analysis of differentially expressed phosphoproteins using STRING. The red and blue halo colors indicate more or less abundant phosphoproteins in the mammary gland of HT (no access to cooling for the ~ 46-day dry period, *n* = 4) compared to CL (cooled with water soakers and fans, for the ~ 46-day dry period, *n* = 4) cows, respectively. (A) 14 days in milk (DIM), (B) 42 DIM, and (C) 84 DIM. The same color of balls indicates those proteins belong to the same cluster based on MCL clustering with a default value in STRING. Enriched biological processes or pathways are shown in the text. The thickness of the edges reflects the strength of connection between the proteins based on known interactions (either inferred from curated databases or experimental results), predicted interactions (gene neighborhood, gene fusion, and gene co-occurrence), and others (text-mining information, co-expression, and protein homology) at medium confidence score. Refer to Supplementary Table S1 for protein ID and its description. Figures were created using STRING (<https://string-db.org>, version 11.0).


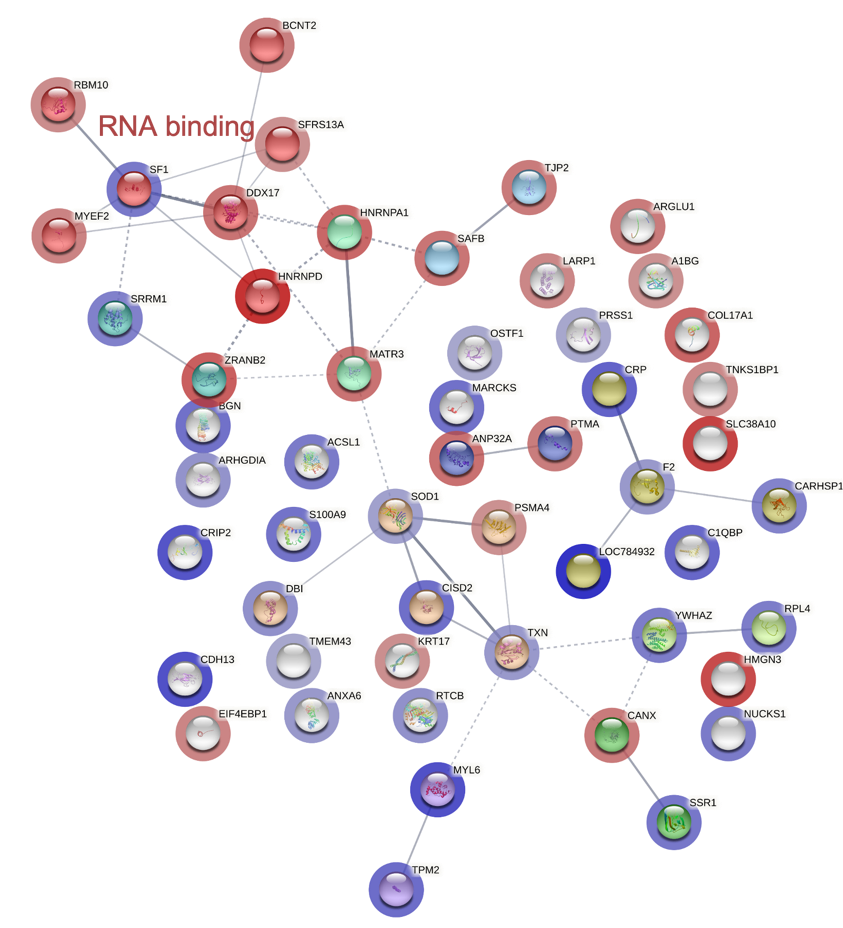
A


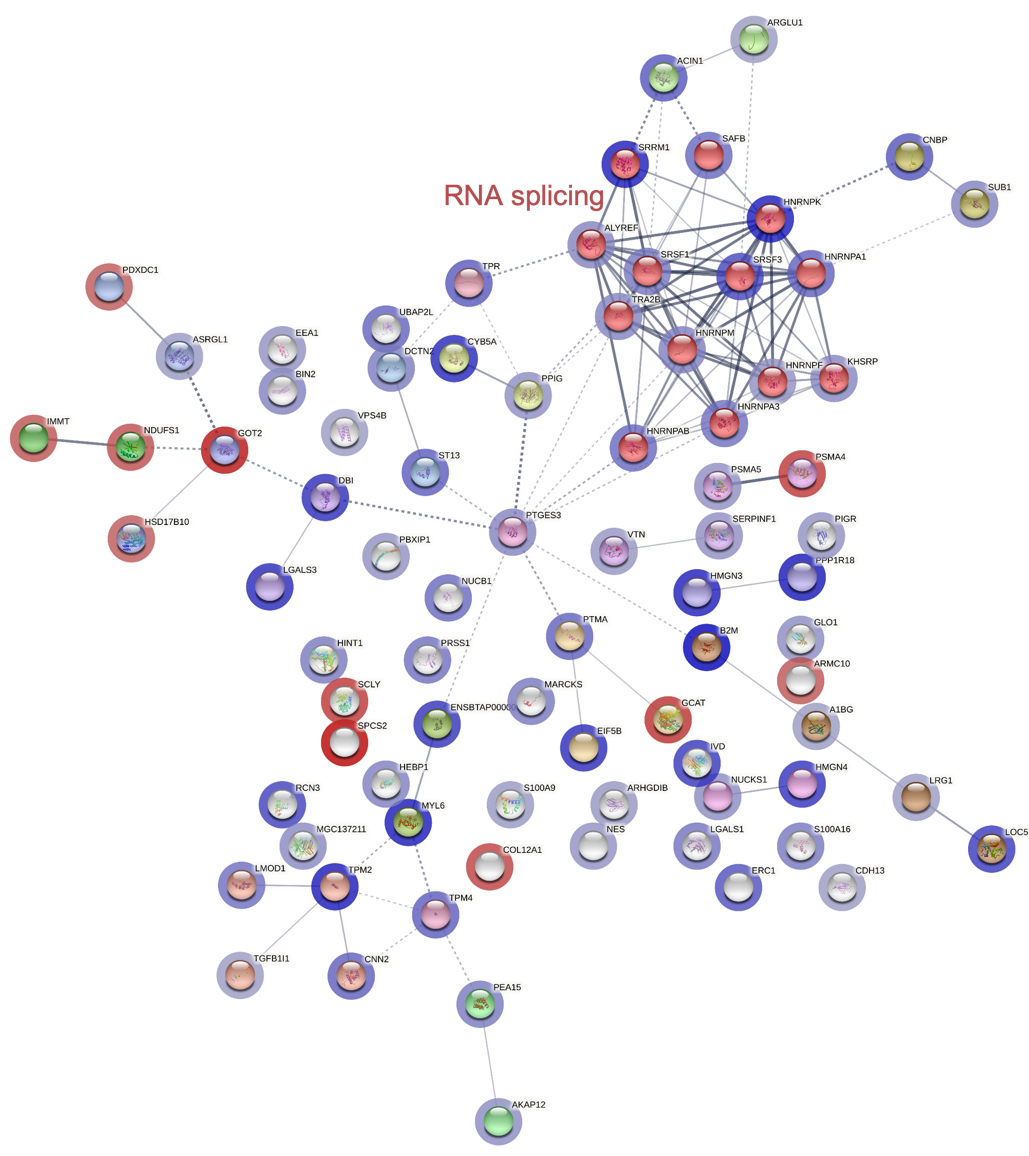
B


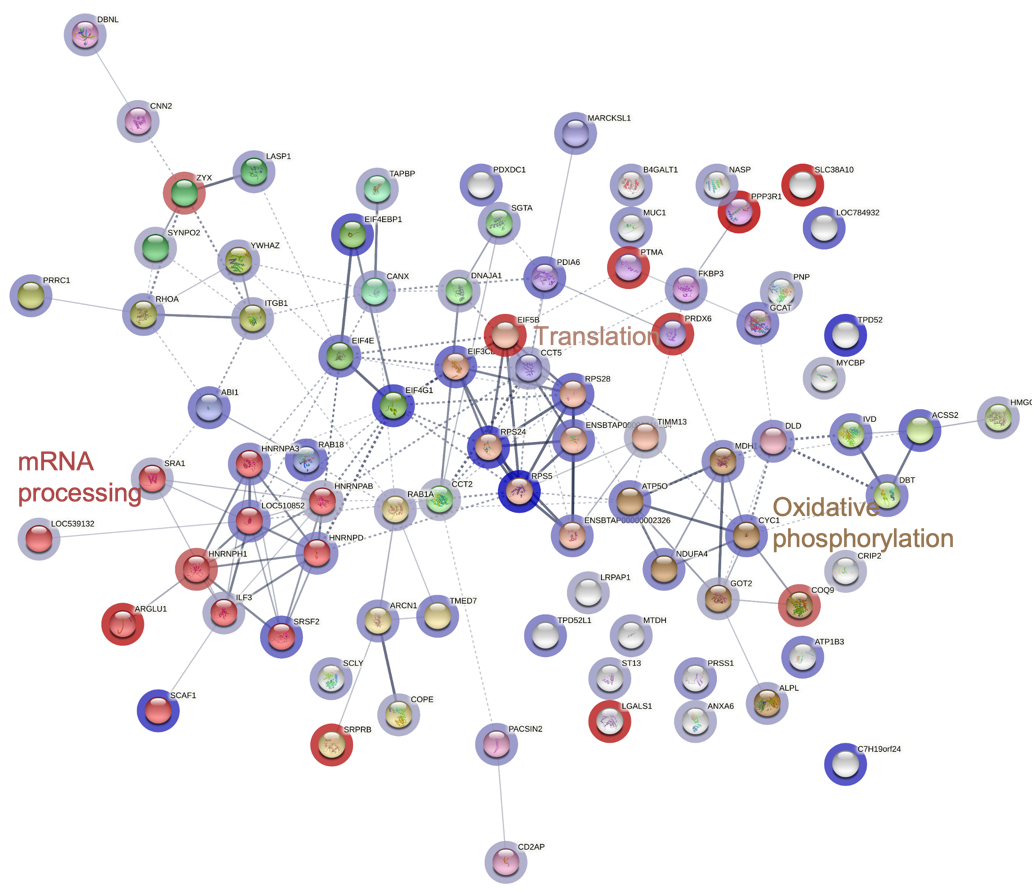
C
